# Rural barriers and facilitators of lung cancer screening program implementation in the veterans health administration: a qualitative study

**DOI:** 10.3389/frhs.2023.1209720

**Published:** 2023-08-22

**Authors:** Jennifer A. Lewis, Kemberlee Bonnet, David G. Schlundt, Susan Byerly, Christopher J. Lindsell, Claudia I. Henschke, David F. Yankelevitz, Sally J. York, Fred Hendler, Robert S. Dittus, Timothy J. Vogus, Sunil Kripalani, Drew Moghanaki, Carolyn M. Audet, Christianne L. Roumie, Lucy B. Spalluto

**Affiliations:** ^1^ Veterans Health Administration-Tennessee Valley Healthcare System, Geriatric Research, Education and Clinical Center (GRECC), Nashville, TN, United States; ^2^ Veterans Health Administration-Tennessee Valley Healthcare System, Medicine Service, Nashville, TN, United States; ^3^ Division of Hematology and Oncology, Department of Medicine, Vanderbilt University Medical Center, Nashville, TN, United States; ^4^ Center for Clinical Quality and Implementation Research, Vanderbilt University Medical Center, Nashville, TN, United States; ^5^ Vanderbilt-Ingram Cancer Center, Nashville, TN, United States; ^6^ Department of Psychology, Vanderbilt University, Nashville, TN, United States; ^7^ Qualitative Research Core, Vanderbilt University Medical Center, Nashville, TN, United States; ^8^ Division of General Internal Medicine and Public Health, Vanderbilt University Medical Center, Nashville, TN, United States; ^9^ Department of Biostatistics, Vanderbilt University School of Medicine, Nashville, TN, United States; ^10^ Department of Radiology, Icahn School of Medicine at Mount Sinai, NY, New York, United States; ^11^ Veterans Health Administration—Phoenix VA Health Care System, Radiology Service, Phoenix, AZ, United States; ^12^ Rex Robley VA Medical Center, Medicine Service, Louisville, KY, United States; ^13^ Owen Graduate School of Management, Vanderbilt University, Nashville, TN, United States; ^14^ Veterans Health Administration—Greater Los Angeles Veterans Affairs Medical Center, Radiation Oncology Service, Los Angeles, CA, United States; ^15^ Department of Radiation Oncology, University of California, Los Angeles, Los Angeles, CA, United States; ^16^ Department of Health Policy, Vanderbilt University Medical Center, Nashville, TN, United States; ^17^ Department of Radiology and Radiological Sciences, Vanderbilt University Medical Center, Nashville, TN, United States

**Keywords:** lung cancer screening, barriers, facilitators, implementation science, low-dose CT, RE-AIM, rural

## Abstract

**Introduction:**

To assess healthcare professionals' perceptions of rural barriers and facilitators of lung cancer screening program implementation in a Veterans Health Administration (VHA) setting through a series of one-on-one interviews with healthcare team members.

**Methods:**

Based on measures developed using Reach Effectiveness Adoption Implementation Maintenance (RE-AIM), we conducted a cross-sectional qualitative study consisting of one-on-one semi-structured telephone interviews with VHA healthcare team members at 10 Veterans Affairs medical centers (VAMCs) between December 2020 and September 2021. An iterative inductive and deductive approach was used for qualitative analysis of interview data, resulting in the development of a conceptual model to depict rural barriers and facilitators of lung cancer screening program implementation.

**Results:**

A total of 30 interviews were completed among staff, providers, and lung cancer screening program directors and a conceptual model of rural barriers and facilitators of lung cancer screening program implementation was developed. Major themes were categorized within institutional and patient environments. Within the institutional environment, participants identified systems-level (patient communication, resource availability, workload), provider-level (attitudes and beliefs, knowledge, skills and capabilities), and external (regional and national networks, incentives) barriers to and facilitators of lung cancer screening program implementation. Within the patient environment, participants revealed patient-level (modifiable vulnerabilities) barriers and facilitators as well as ecological modifiers (community) that influence screening behavior.

**Discussion:**

Understanding rural barriers to and facilitators of lung cancer screening program implementation as perceived by healthcare team members points to opportunities and approaches for improving lung cancer screening reach, implementation and effectiveness in VHA rural settings.

## Introduction

Lung cancer screening with low-dose computed tomography (LDCT) in high-risk individuals is an evidence-based practice that improves lung cancer mortality (1, 2). It is recommended by the U.S. Preventive Services Task Force for individuals between the ages of 50 and 80 who have a tobacco history of at least 20 pack-years and currently or formerly smoked and quit within the past 15 years (3). It is also covered by Medicare in a similar patient population and is recommended by many professional organizations (4). Yet, while lung cancer screening is widely recommended, uptake in clinical practice is exceedingly low (5, 6), including in Veterans Health Administration (VHA) (7). Furthermore, approximately one quarter of Veterans reside in rural communities (8), and rural Veterans are less likely to undergo initial and subsequent lung cancer screening (9). The gap in effectively implementing this evidence-based practice may lead to missed opportunities to identify lung cancer early and improve patient health outcomes.

Translation of evidence-based practices to regular clinical practice is often delayed by many years (10, 11). Rigorous implementation science approaches can aid in expediting the translation of evidence-based practices such as lung cancer screening into regular clinical care to improve patient outcomes (12, 13). Understanding healthcare team members' perception of rural barriers and facilitators to implementation of lung cancer screening programs in VHA is an important initial step in developing an implementation science-driven approach to support high quality lung cancer screening that reaches all Veterans.

The objective of this study was to assess VHA radiology and primary care healthcare team members' perceptions of rural barriers and facilitators of lung cancer screening implementation through a series of one-on-one interviews. This study is embedded within a larger parent study, the Veterans Affairs Partnership to increase Access to Lung Screening (VA-PALS) Program Evaluation, that uses the Reach, Effectiveness, Adoption, Implementation, Maintenance (RE-AIM) framework to develop measures for evaluating lung cancer screening program implementation across 10 Veterans Affairs medical centers (VAMCs) (14).

## Materials and methods

### Study design and data sources

We conducted a cross-sectional qualitative study consisting of one-on-one semi-structured phone interviews with healthcare team members. Interviews lasted up to 1-hour. The findings of this study are reported following the guidelines of the Consolidated Criteria for Reporting Qualitative Studies, an evidence-based qualitative methodology (15). Agreement was obtained via telephone and documented.

This study was reviewed and approved by the Veterans Affairs (VA) Central Institutional Review Board (C-IRB E19-05), VA Tennessee Valley Healthcare System Research & Development Committee, the VA Organizational Assessment Subcommittee, and the VA Office of Labor and Management Relations (national union approval). The decision to publish was made by the study team.

### Theoretical frameworks

We utilized the RE-AIM framework to develop VA-PALS Program Evaluation measures (16). This evaluation framework, which has been used in the cancer screening setting (17), provides a comprehensive structure to assess real-world clinical programs. The current study provides important measures within the “Implementation” domain of RE-AIM. This domain refers to whether an intervention or evidence-based practice was delivered with fidelity, or as originally intended (16). We also used the Consolidated Framework for Implementation Research (CFIR) in planning the overall program evaluation to understand contextual factors (e.g., team members, inner settings, processes at each VAMC) of lung cancer screening program implementation and the FRAME framework to study adaptations in lung cancer screening delivery (18, 19).

### Recruitment and study population

Study inclusion criteria included healthcare team members [clinicians and staff (nurses, technologists, schedulers, administrative leaders)] and program directors employed at VAMCs participating in VA-PALS (Atlanta, Chicago-Hines, Cleveland, Denver, Indianapolis, Milwaukee, Nashville, Philadelphia, Phoenix, St. Louis). In this convenience sample, we recruited participants via email who expressed interest in continued research participation during a previous VA-PALS survey (20) and also recruited participants via champions who disseminated study information. Direct participant remuneration was not offered due to VHA policy. An initial recruitment goal of 30 participants was estimated. Study recruitment was stopped when thematic saturation was reached and no new emerging themes were identified.

### Interviews

Semi-structured interview guides were tested, modified, and finalized by conducting pilot interviews with study team members. Participants were asked questions relating to: (1) employment history; (2) percentage of clinical practice residing in rural areas; (3) attitudes and beliefs about lung cancer screening; (4) lung cancer screening delivery processes; (5) roles and responsibilities; (6) teamwork and interprofessional communication; and (7) barriers and facilitators to implementation and screening. For staff and administrative professionals, questions pertaining to clinical decision-making were not asked. For participants whose facility had not yet implemented a lung cancer screening program, the questions were presented in a hypothetical frame. The interview guide consisted of additional questions for program directors relating to: (1) program team characteristics; (2) degree of leadership buy-in; (3) alignment with Veterans Integrated Service Network (VISN) priorities; (4) navigator retention; (5) navigator work length in days; and 6) establishment of navigators in permanent position (Supplementary Appendix S1).

### Data collection

Study personnel contacted potential participants to schedule an interview date via email. Prior to the interview, participant agreement was obtained by telephone. Interviews were conducted via telephone in a secured office at the Nashville VAMC. Only the interviewer and interviewee were present. Each interview was conducted by a trained, female, MA psychologist (KB) using the appropriate interview guide for type of participant. Follow-up questions were asked to confirm understanding and facilitate more detailed discussion. Field notes were not made. The interviewer did not have a relationship with any of the participants prior to the interview. Participants were aware of the study's purpose to understand implementation of lung cancer screening in their VAMC and that the interviewer was a trained qualitative researcher without a clinical background in lung cancer screening. The audio-recorded interviews were transcribed and de-identified by a transcriptionist. Transcripts were not reviewed by participants and repeat interviews were not performed. Study personnel maintained recordings and study activity logs on a password-protected computer housed in a locked office at the Nashville VAMC. Each participant was assigned a unique participant identification number.

### Qualitative data coding

Qualitative data coding and analysis was managed by the Vanderbilt University Qualitative Research Core, led by a MA psychologist (KB). A hierarchical coding system was derived inductively with preliminary review of the transcripts and deductively using the interview guides and CFIR (18). Major coding categories included (1) screening activity; (2) lung cancer screening program characteristics; (3) hospital organizational setting; (4) outer setting; (5) intervention characteristics; (6) patient factors; (7) communication; (8) barriers and facilitators; (9) specific examples; (10) process; (11) suggestions and needs; (12) provider/health team member; (13) practice/work experience; (14) world events; (15) change over time; and (16) notable quotes. Major coding categories were further divided from one to 13 subcategories, with some subcategories having additional levels of hierarchical division. Definitions and rules were established for the use of coding categories (Supplementary Appendix S2).

Three experienced qualitative coders independently coded two transcripts from each type of participant (provider, staff, program director). Coding was then compared, and any discrepancies resolved by reconciliation. Coders divided and independently coded the remaining transcripts. Each statement was treated as a separate quote and could be assigned up to 24 distinct codes (major code categories plus sub-categories). The coded transcripts were combined and sorted by code. Transcripts, quotations, and codes were managed using Microsoft Excel 2016 and SPSS version 28.0.

### Conceptual model development

We used an iterative inductive deductive approach to the qualitative analysis, which resulted in development of a conceptual model (21–23). Deductively, the model was guided by CFIR at the institution and systems level, and at the patient level by the theory of Self-Care Management for Vulnerable Populations, the Biopsychosocial Framework, and the Social Ecological Model (24–26). Inductively, the details of the conceptual model were informed by the themes identified in the coded data.

## Results

### Study participants and demographics

Of 54 participants invited, five were not interested, in-eligible, or unable to participate, and 19 have unknown reasons for not participating. A total of 30 completed an interview between December 2020 and September 2021. Data from these 30 interviews comprised the final analytic sample. Table 1 demonstrates the characteristics of healthcare team members participating in an interview, including VA site, self-identified professional training, specialty, time in specialty, employment within the past 5 years, program director role, and perceptions of the proportion of rural patients served in clinical practice. Providers included oncology, primary care, pulmonology and radiology physicians, advanced practice providers, and staff.

**Table 1:** Characteristics of interview participants.

Characteristic	*n* (%), *N* = 30
VA Site	
Atlanta	1 (3)
Chicago-Hines	2 (7)
Cleveland	3 (10)
Denver	3 (10)
Indianapolis	3 (10)
Milwaukee	3 (10)
Nashville	3 (10)
Philadelphia	5 (17)
Phoenix	3 (10)
St. Louis	4 (13)
Specialty	
Oncology	2 (7)
Primary Care	10 (33)
Pulmonology	7 (23)
Radiology	7 (23)
Other or Not applicable^a^	4 (13)
Professional Training	
Physician	14 (47)
Advanced Practice Provider	3 (10)
Nurse	6 (20)
Technician/Technologist	4 (13)
Administrative Support Assistant	2 (7)
Other^b^	1 (3)
Time in Role	
1–10 years	11 (37)
11–20 years	10 (33)
>20 years	9 (30)
Employment in past 5 years	
Practiced within VA	20 (67)
Practice within and outside VA	10 (33)
Leadership	
Lung Cancer Screening Program Director	10 (33)
Perception of clinical practice that is rural	
≤10%	2 (7)
11%–25%	7 (23)
26%–50%	9 (30)
51%–75%	2 (7)
>76%	1 (3)
Missing^c^	9 (30)

^a^ Some participants were in Tobacco Cessation outside of Primary Care, Radiology, Oncology or Pulmonology or in a Medical support Assistant role that did not align with a particular specialty.

^b^ One participant was in a program analyst role.

^c^ Some participants were unsure of percentage of rural Veterans in their practice or this was not a part of their healthcare role.

The duration of interviews ranged from 18.23 to 66.29 min (median 41.39 min; IQR:32.08–51.11 min).

### Conceptual model for rural barriers and facilitators of lung cancer screening

**Figure 1:**
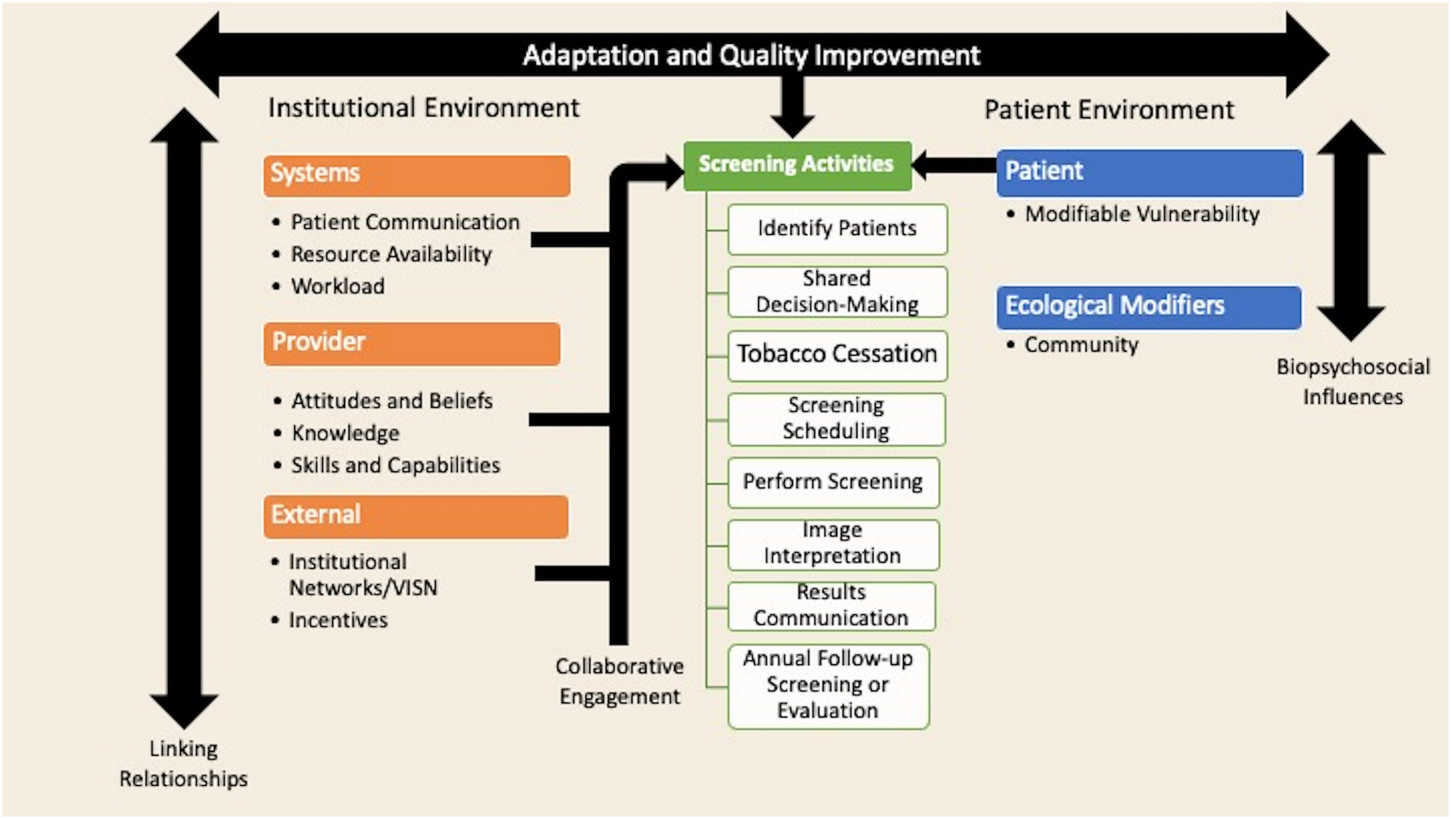
Conceptual model of rural barriers and facilitators of lung cancer screening implementation. This model was guided deductively by the Consolidated Framework for Implementation Research at the institution and systems level, and at the patient level by the theory of Self-Care Management for Vulnerable Populations, the Biopsychosocial Framework, and the Social Ecological Model (24–26). This model was guided inductively by the themes identified in the coded data.

Figure 1 presents a conceptual model that organizes the qualitative results. The model has three main elements: (1) the institutional environment, (2) screening activities, and (3) the patient environment. There is bi-directional influence of both the institutional and patient environments that influences the planning and implementation of the screening elements. Healthcare professionals within local and the larger healthcare system develop linking relationships that can either facilitate or hinder the implementation of screening programs. These linking relationships, at the local systems and provider level, lead to collaborative engagement that can facilitate implementation of the screening programs. Within the patient environment, the full range of biological, psychological, social, and cultural influences interact to influence a patient's willingness to engage in screening. Within this model, there are numerous opportunities for interruption of the screening process. Adjustments to the process through adaptation and quality improvement can enhance the screening workflow processes.

We use this overall model to discuss major and subthemes of rural barriers to and facilitators of lung cancer screening program implementation, which is summarized in Table 2. We use supporting quotes from participants. Each quote identifies the participant study identification number and healthcare team role.

**Table 2:** Summary of rural barriers and facilitators of lung cancer screening Implementation.

Environmental context	Level of influence	Type of influence	Barrier or facilitator	Example from qualitative data
Institutional environment	Systems	Patient communication	Facilitator	Use of language common to rural communities during lung cancer screening shared-decision making conversations Shared decision-making training tailored to rural Veterans who currently smoke and may fear screening results
		Resource availability	Facilitator	Use of tool to manage patients being screening to ensure future care is coordinated Mobile CT screening or screening done at community clinics (CBOCs)
			Barrier	Lack of access to rural clinics
		Workload	Barrier	High personnel turnover Lower priority among healthcare services
	Provider	Attitudes and beliefs	Barrier	Lack of centralized and specialized care for cancer screening and treatment
		Knowledge	Barrier	Lack of knowledge of screening program activities and/or importance
		Skills and capabilities	Barrier	Time delays in screening and coordination of care in rural settings Lack of centralized screening program that manages and coordinates care Lack of radiologist use of standardized reporting system for lung cancer screening results
	External	Institutional networks	Facilitator	Regional educational programs by specialists
		Incentives	Facilitator	Offering monetary incentives for referring for screening
Patient environment	Patient	Modifiable vulnerabilities	Barrier	Patient need for transportation Poor living conditions Lack of education
			Facilitator	Development of telephone or video clinic Scheduling screening on day when patient is already coming to the medical center or area Lung cancer screening education specific for rural patients Mailings to increase awareness of lung cancer screening among rural patients
	Ecological modifiers	Community	Barrier	Lack of discussions on health behaviors in rural communities Misinformation Rural norms and culture may not focus on some healthcare practices

Data obtained in 2020 to 2021 from interviews with radiology and primary care staff and providers and lung cancer screening program directors at 10 VAs participating in an enterprise-wide initiative, VA partnership to increase access to lung screening (VA-PALS); CBOC stands for community based outpatient clinic.

## Institutional environment

### Systems

#### Patient communication

Communities often have shared language and expressions. This participant describes using language common among rural Veterans during lung cancer screening shared decision-making to ensure comprehension:

*“So when you communicate with shared decision making, you’re making sure that you are speaking to them in terms that they understand and can relate to and can appreciate and that you’re not really speaking over them or giving complex concepts or thoughts that they may not really understand.” (Participant 16, Program Director)*

Some patients who are eligible for lung cancer screening currently smoke cigarettes and may or may not want to quit smoking. This participant describes needing assistance to communicate with rural patients who currently smoke with no plans to quit smoking and may have a fear of knowing whether they have lung cancer:

*“I think some of them just feel like if they’re not going to quit, I mean so many people are just like I’m going to smoke. So, I don’t think they care about knowing but I’ve never had conversations like are you afraid to know. Yeah maybe some guidance on what those conversations on what those conversations should look like. You know they kind of are judgmental, leaning to some judgment …”. (Participant 8, Nurse Practitioner)*

#### Resource availability

Lung cancer screening programs increase screening reach and sustainability with resources such as personnel (patient navigators) and access to CT scanners, tobacco cessation resources, and specialists in cancer care (27). One participant explained that rural access to care is a barrier:

*“The VA doesn’t have a lot of contracts with really rural areas. So, if they’re going to go see mental health, to deal with their depression, anxiety, weight, smoking or whatever, it's still access. Finding somebody close to you who's taking VA payments and honestly a lot of our patients just kind of give up. Because it's easier to give up and keep smoking than it is to address it.” (Participant 7, Nurse Practitioner)*

Participants highlighted the possibility of mobile CT screening, the need for a tool to help manage screening done in rural settings, and access to radiologists who have expertise in interpreting lung cancer screening imaging:

*“We have one satellite that does it but it's also within 15 miles of the main medical center so the people that have declined being a part of the program almost always its because they don't want to get their scans done here. So, one of the things that I think will be a great idea moving forward is going to be some sort of mobile scanner. If you have a CAT scan on a truck and you can move it to the different areas, you're going to have a much more successful program.” (Participant 20, Program Director)*

*“I would love it if we had some way to have the screens done the right way either a mobile or local CBOC being able to take the CT the right way with low dose and get it sent to our radiologists that actually have experience reading it and then we can follow it up. The problem is we have no way to track those. We have no way to get the images.” (Participant 19, Program Director)*

#### Workload

Participants discussed challenges related to rural provider workload. Rural facilities disproportionatly experience high provider turnover, due to limited resources and high workloads. This participant describes rural provider turnover and burnout as a challenge to maintaining a consistent screening program:

*“… our rural clinics and our rural CBOCs tend to have a fairly large turnover in providers. So, I think that's a challenge, right? You know there's kind of a churn and burn and so I think some rural providers this may be lower on their list in the sense of what it requires.” (Participant 17, Program Director)*

Providers manage multiple health care needs and problems for patients. Lung cancer screening may be less of a priority in the rural setting due to high workload:

“Rural meaning you’re spread out and they’re fewer of you and therefore it's more difficult to get consistency and to get buy in. It's hard to remember that the objective is to drain the swamp when you’re up to your [a**] in alligators. You take care of all sorts of stuff and wait a minute, ‘I don’t want to make more work for myself. I’ve got enough to do.’ So, I understand the problems involved …” (Participant 5, Physician)

### Provider

#### Attitudes and beliefs

Several participants discussed their perceptions of healthcare in rural areas. These participants pointed out that surgery for cancer at academic medical centers is highly specialized and may not be available in rural communities:

*“Some of the patients don’t understand that having cancer care or having surgery for stage 1 lung cancer being done by board certified thoracic surgeon is better than having surgery done by a general surgeon. So, having cancer care provided in an academic affiliated facility, and I’m biased in my statement, is probably better than some of the care than this patient would receive in their rural area.” (Participant 15, Program Director)*

*“Our belief here is that you know when you refer someone out for lung cancer screening with the mission act, its complicated and it's scattered care. I usually tell the patients you’re better off coming here. If we really have to do something where you live we’ll do it but we also want to have you within the healthcare system where we’re familiar with you and we can access your medical record, those kind of things.” (Participant 17, Program Director)*

#### Knowledge

Lung cancer screening is a complex process that involves shared decision-making, tobacco cessation, yearly follow-up screenings, and coordination of care to evaluate and treat cancer (e.g., pulmonology, oncology, thoracic surgery, radiation oncology). Programs dedicated to lung cancer screening help track when Veterans are due for their next screening or need evaluation for possible cancer. This participant describes lack of provider knowledge of screening programs in rural areas:

*“… If my Veteran has to drive down to our VA which takes an hour vs. an hour or two hours away to somewhere else, we usually just counsel them that hey you can’t just get it at your local place. Just come down here and get the scan and we’ll take care of it. So that's one thing and its hard to because I don’t think all primary care physicians particularly those in CBOCs who are seeing most of these rural Veterans completely get that either. So, we have tried to do outreach and education, but it is hard because its kind of a different way of looking at it.” (Participant 19, Program Director)*

#### Skills and capabilities

This participant discussed delays associated with getting imaging done in rural communities and that imaging results do not use a standardized reporting system that informs healthcare providers of the next step in lung cancer screening care (i.e., results needing immediate work up, repeat early screening or repeat screening in 12 months).

*“… Some of them don’t want to come to the VA to have the CT scan done so they get their LDCT done in a community imaging center and we can coordinate for that but it is sometimes a little bit tricky and more difficult to get those reports back into the VA. So there maybe a little bit of delay there. And associated with getting that report from a community imaging center sometimes the challenges that the report is not in the format that we want the data reported … that is what we call structure report …” (Participant 24, Program Director)*

Participant expressed that rural hospitals lack lung cancer screening programs:

*“… I’m a big firm believer that lung cancer screening is more than a CT scan. You cannot do lung cancer screening by just sending a Veteran to their local VA or their local hospital through the Mission Act and just getting a CT. It just doesn’t work …So its not helping our rural Veterans that much to have something like the Mission Act because they can go somewhere else but if they’re actually going to get the advantage of lung cancer screening and go to a screening program most of those are in urban areas.” (Participant 19, Program Director)*

### External

#### Institutional networks

VAs are organized into regional networks called Veterans Integrated Service Networks, or VISNs. This participant emphasizes the opportunity for on-site presentations to rural providers within their VISN:

*“_____ is a specialty care access network, so it is a way for people in pulmonary and other specialties to give talks to providers that are located in CBOCs and smaller hospitals across our whole VISN, across the nation really. So, I think some of those providers out there love it because they don’t, you know …. there's talks all day for me around here but there's much fewer opportunities at the more rural settings. So, I think being able to put a name with a face and present cases or present guidelines or present plans is super helpful …” (Participant 17, Program Director)*

#### Incentives

Reinforcement and incentives can be used to encourage provider behavior. This participant suggested that a nominal monetary incentive could help create rural provider buy-in:

*“I think what you’re trying to do is taking a burden off the local practitioner and say all you have to do is send them into this study …That's going to get you some buy in. Particularly if they get a set amount of money to screen. Not to get people in because then you’ll have a bias of them just throwing it to people because they get money out of it. But if they get money for doing so many screenings screening that gets into the program and you get the money for doing that as part of your screening. You get five extra dollars for every time you do a H&P …” (Participant 5, Physician)*

## Patient environment

### Patient

#### Modifiable vulnerabilities

One participant describes their program's approach to rural patient transportation challenges by developing a telephone clinic, as the Veteran can engage in elements of the lung cancer screening program remotely:

*“So, the biggest challenge with the rural Veterans was transportation. We took care of that with our telephone clinic. So, the only time that we ask them to come to the VA is to get the CT scanning done.” (Participant 24, Program Director)*

This participant suggested that another strategy to overcoming transportation challenges is to schedule screening appointments in conjunction with other medical care:

*“Like when they come in for podiatry okay let's see if we can get it scheduled that day you know. Or like schedule your scan for this day and then we’ll let the clinic know and can they get you in that day too …So that helps some of the Veterans know like okay they can come in and get something else …That two-hour drive was worthwhile to come into town and do other errands while you’re here.” (Participant 13, Registered Nurse)*

Participants noticed that some rural Veterans live in poor housing conditions:

*“You know a lot a patients live in houses we would never dream of living in. Like our patients live in burned out mobile homes or other things where they’d be bad places. Home health can’t go out to him until he gets his house cleaned ….Right now, the main thing is the mouse infestation for him. But again, he continues smoking. Even if I do a CT of his chest and find something there, I seriously doubt if he's going to do anything about it.” (Participant 7, Nurse Practitioner)*

This participant suggested the need for specific education for rural Veterans.

*“I wonder if there's maybe sometimes a little more education that needs to be involved with the rural patients … most of them are like farmers or they don’t have jobs that they needed to go to college for. So maybe a lack of education with the rural Veterans maybe there.” (Participant 1, Registered Nurse)*

When asked about the best way to inform rural Veterans about the screening program, this participant suggested mailings:

* “I think probably a mailing. I’ve been thinking about like my 80-year-old patient that I talked to the other day, he was saying he doesn’t have a computer or an iPad or anything, so I think rural patients are a lot like that too. They don’t always have technology but yeah, maybe sending a letter.”(Participant 8, Nurse Practitioner)*

### Ecological modifiers

#### Community

Community can influence health behaviors. This participant suggests that health behaviors may not be a topic of discussion in rural communities:

*“I mean just sometimes they are being out in the country without a lot of neighbors and you know, being part of a small community where you know, health care may not be what people talk about in a rural setting. The awareness factor would just be a barrier.” (Participant 1, Registered Nurse)*

This participant perceives that rural Veterans may obtain information from their community. This information could be inaccurate and deter them from participating in programs:

*“… Cause you know the lady down the road didn’t have a lot of interaction and everybody knows everybody in some respect and in some areas just aren’t getting the right information. They’re getting it from their neighbor you know or from the guy at the store that they go to or a relative.” (Participant 13, Registered Nurse)*

Community norms and culture are important contextual factors to consider in implementation of evidence-based practices. This was highlighted when one participant brought up cultural factors related to tobacco use and lung cancer screening among rural Veterans:

*“Almost every one of them either smoke or dip and have their whole lives because they live on farms. I mean that's what they do. They’re country people and if they didn’t do it, their grandparents did it, so they do it. It's part of them.” (Participant 6, Licensed Practical Nurse)*

Another participant perceived that rural communities may have their own healthcare practices or norms:

“*Well you know they have their own way of life. Things are quiet and don’t run the flow. You know they’re just kind of simple or you know have different ways of life or styles of what or how you take care of this and you know just going to the ….Oh back in my day in home care like they have their own family way of taking care of this problem or the things they would take or apply to themselves was very interesting.” (Participant 13, Registered Nurse)*

## Discussion

This study is among the first to describe VHA healthcare team members’ perceptions of barriers and facilitators of lung cancer screening program implementation in the rural setting. Systems-level barriers and facilitators included patient communication, resource availability, and workload. Provider-level barriers and facilitators included provider attitudes and beliefs, knowledge, and skills and capabilities. External to systems, institutional networks and incentives were discussed as facilitators. Perceived barriers and facilitators at the patient-level included modifiable vulnerabilities and the rural community was found to be an ecological modifier.

Commonly perceived barriers to implementation of new practices include lack of resources, staff shortage and turnover, lack of leadership buy-in, organizational culture, lack of expertise, and challenges with adapting the implementation of evidence-based practices (28, 29). A study by Gesthalter et al. compared lung cancer screening program implementation among three VAMCs that were early to adopt lung cancer screening. The study team conducted in-depth interviews with programmatic staff from 2013 to 2014 and found that managing workloads and obtaining primary care buy-in were major barriers (30). We found similar barriers in the need to manage workloads. For example, one participant described the need for a tracking system to help manage patients screened in rural communities and the need for specialized cancer screening and treatment care. However, the present study uniquely highlights perceived barriers at the patient-level. We discovered themes related to the need to tailor communication to rural Veterans, difficulty with transportation, and the potential influence rural communities may have on Veteran screening behavior. These findings add to the existing literature by highlighting areas for future lung cancer screening program growth and intervention development to increase rural screening reach and sustainability.

Improving the implementation of lung cancer screening in clinical practice offers an opportunity to not only improve overall lung cancer morbidity and mortality outcomes, but also to address existing disparities, such as improving lung cancer outcomes for Veterans living in rural areas. We found perceived barriers particular to rural Veterans included lack of resources, communication differences, transportation challenges, lack of clinics in rural communities, lack of lung cancer screening programs, provider knowledge gaps, and time delays in coordination of screening-related care. Several barriers found in our study, including provider lack of knowledge, geographic distance and transportation, and a lack of screening programs, are similar to those found in rural Colorado. In this study, researchers determined that a lack of a team-based and systematic and/or automated approach to conducting lung cancer screening resulted in few offers of lung cancer screening (31). Rural barriers found in the current study are similar to those of breast, cervical and colorectal cancer screenings and among rural populations (32–36). These include difficulty accessing care due to structural barriers such as travel distance and transportation challenges as well as shortages of healthcare providers (34). The implementation of lung cancer screening programs with virtual clinic options in combination with mobile CT units that travel to rural areas may help to offset some of these challenges.

Potential strategies found in the present study to address rural disparities included education specific to rural Veterans, training in how to tailor communication to rural Veterans during shared decision-making, mobile lung cancer screening, and bundling screening with other appointments or activities closer to screening centers. The Community Services Preventive Task Force provides recommendations to increase breast, cervical and colorectal cancer screenings: provider audit and feedback (breast, cervical and colorectal), one-on-one education and patient reminders (breast and cervical), one-on-one education (colorectal) and group education (breast) (32). A recent review of the literature on rural communities found that individual and group education successfully increased breast and cervical cancer screening but that testing of strategies to increase patient adherence to yearly breast and cervical cancer screening were lacking (36). The use of a patient navigator may be a strategy to increase lung cancer screening adherence as well as breast, cervical and colorectal cancer screening (37). Further work is needed in this area.

VHA is dedicated to lung cancer screening, diagnosis, and treatment and has supported multiple initiatives over the last decade (14, 38, 39). With the goal of reaching rural Veterans, VHA uses virtual clinics (telephone and video) to screen Veterans, and we expect these services to remain in place to increase and sustain rural screening reach (19). VHA will be starting mobile lung cancer screening as part of the Lung Precision Oncology Program. This program started in 2021 with the following goals: (1) prioritizing screening to identify early-stage lung cancer; (2) offering genetic testing for treatment of advanced stage lung cancer; (3) improving access to precision-oncology clinical trials for treatment of advanced stage lung cancer; (4) increasing availability of clinical trials to provide new treatment options for lung cancer and (5) enabling rapid translation of discoveries into clinical care (39).

The use of established implementation science frameworks greatly strengthened this qualitative research study. Our study team leveraged the RE-AIM framework to develop the overall program evaluation for the VA-PALS parent study (14). Guided by “Implementation” domain in the RE-AIM framework, we designed this qualitative study of in-depth interviews with healthcare team members on the frontlines and in leadership positions to assess barriers and facilitators of lung cancer screening program implementation. Complementary research has demonstrated the use of RE-AIM to evaluate rural implementation barriers and facilitators of lung cancer screening in Colorado. In this qualitative study among rural primary care team members (clinicians, staff, patients) in multiple clinical settings (federally qualified health centers, private practices, and health system owned clinics), investigators used RE-AIM for data analysis and to process diagram how decisions impacted the different RE-AIM domains (i.e., Reach, Effectiveness, Adoption, Implementation, Maintenance) (31). In another study, a VA team has used RE-AIM to guide its implementation outcomes in a hybrid-effectiveness study that will test a lung cancer screening shared decision-making tool in New England VAs (40). Our study is unique in its application of RE-AIM to VHA program evaluation of lung cancer screening. Differing from the prior study by Gomes et al., we included radiology as well as primary care team members as well as program directors but did not include patients. This study also leveraged the expertise of an established qualitative research team, and qualitative study design that increases understanding of individual perspectives of rural barriers and facilitators to implementation of lung cancer screening and strategies to overcome these barriers.

Study limitations include our convenience sample of recruited participants via survey and champions rather than random selection, which may have introduced selection bias. Further, we do not have full demographic data (age, gender, race, ethnicity) on the overall characteristics of the healthcare providers nor those who declined an interview to allow for demographic characterization of responders and non-responders, which may introduce non-responder bias.

Understanding the barriers and facilitators of lung cancer screening program implementation in the rural setting as perceived by healthcare team members offers an opportunity to improve lung cancer outcomes while reducing disparities among the Veteran population. Our results may inform development and testing of strategies to improve lung cancer screening program reach, effectiveness and adoption.

### Article Summary

Identifying rural barriers and facilitators of lung cancer screening program implementation in the Veterans Health Administration offers an opportunity to understand and improve rural-urban disparities and delivery of care for Veterans at high-risk for lung cancer.

## Data Availability

The dataset used in the present study is available from the corresponding author upon reasonable request.
