# Supplementary material for: Rural barriers and facilitators of lung cancer screening program implementation in the veterans health administration: a qualitative study
Source: Front Health Serv. 2023 Aug 22;3:1209720. doi: 10.3389/frhs.2023.1209720 (PMC10477991; doi:10.3389/frhs.2023.1209720)
Supplement: Supplementary file 1 [file Datasheet1.docx]

Supplementary Appendix S1

Interview Guide for Evaluation of Barriers to and Facilitators of Lung Cancer Screening Program Implementation

Project Title: Implementation of Lung Cancer Screening in the VA

Method: In-depth Interviews

Target Audience: radiologists, primary care providers (MD/APPs), staff (plan to target primary care schedulers, radiology technicians), administrative leaders

Supplies: 4 recorders, batteries, 2 notepads, 6 pens, 14 consent forms

Maximum participant time required: 1.0 hr

Introduction (10 minutes)

Thank you for agreeing to an interview today. My name is ________ and I am from Nashville VA. We are conducting research on the implementation of low-dose computed tomography (low-dose CT) for lung cancer screening (LCS) at your VA. We feel that it is very important to speak directly to you about your experiences. Therefore, we will be conducting interviews with VA providers and staff in the areas of primary care and radiology.

During the interview today, we will be asking about your experiences and thoughts on cancer screening, focused primarily on lung cancer screening. We are most interested to hear about your own personal experiences, thoughts, and opinions of the new lung cancer screening test, low-dose CT. So, please do not feel shy. Your views are extremely valuable to us, and we are here to learn from you. I have a list of topics I would like to discuss with you, but feel free to bring up any topics you feel are related to the discussion. Also, I want to let you know that your participation in this interview is completely voluntary. If you want to stop at any time or don’t feel comfortable answering a question, please let me know.

I would like to record our discussion so that the rest of the research team can also hear your views exactly and we don’t miss anything you say. Our discussion will remain confidential; only the research team will listen to the recording and the information you give will only be used for this research project to improve lung cancer screening implementation in the VA. Is it ok to record the discussion? Our interview will last about one hour. Are there any questions before we start? (consent form completed prior to telephone interview)

Warm Up (10 minutes)

1. **How long have you been a __________(provider/staff/administrator) in _________ (specialty field)?**

2. **Where else have you practiced/worked in the past 5 years?**

**-VA and non-VA**

3. **What percentage of your clinical practice live in a rural area?** *(primary care providers only)*

**Do you have a sense of how many Veterans at your VA live in rural areas?** *(radiologists, staff/admin)*

4. **Tell me about your thoughts on lung cancer screening?**

Do you think it is worthwhile? Will it benefit patients? Is it valuable?

Is there evidence that lung cancer screening saves lives from lung cancer? *(providers only)*

Is lung cancer screening a priority for you? *(primary care providers only)*

Is lung cancer screening a priority for your radiology service? *(radiology providers only)*

Main Questions (40 minutes)

*(Read the screening process below for the participant)*

Lung Cancer Screening Involves the Following Activities:

1. Identification of patients who are appropriate for screening (older persons, heavy smokers)

2. Providers and patients discuss the benefits and risks of screening (shared decision-making)

3. Provider orders screening test or refers the patient for screening

4. Appointments for smoking cessation and screening are scheduled

5. Smoking cessation services offered

6. Screening study (CT scan) is performed

7. Radiologist interprets the screening study

8. Screening results are sent to the referring provider

9. Referring provider discusses the screening results with the patient

10. Results are tracked over time in an electronic software system

11. Annual screening follow up or further evaluation is scheduled

5. **Tell me about your role in lung cancer screening.**

Were you involved in or are you currently involved in the decision to implement a lung cancer screening program at your VA?

Describe the process your VA went through to start a lung screening program. If you are currently in the decision-making process, what considerations have come up?

Who would be responsible for ensuring that the lung cancer screening is implemented correctly and consistently over time?

6. **What has been difficult in performing lung cancer screening?** *(ask everyone)*

Possible answers: Identifying eligible individuals, performing shared decision making (discussing potential risks/benefits) [who is performing the shared decision making with the veterans- any difficulties with that?], who shares the results with Veterans- who is following up on screening results, knowing what next steps to take based on screening results [combo of staff and veteran], identifying those who need a repeat screening in 3,6, or 12 months; patients are not motivated, patients cannot afford screening, leadership does not support screening [service level or executive leadership level]

7. *Program Directors Only*: **Tell me about the lung cancer screening program at your VA**.

- Who is on the team (specialties represented (i.e. radiology, pulmonology, primary care, oncology, surgery, etc.; types of providers such as physicians, nurse practitioners/physician assistants, or nurses)

- How many navigators are on the team? Have there been previous navigators? How many days a week do the navigator(s) work for the lung cancer screening program?

- Describe how the team works together on a daily basis? How does the team communicate? Handle challenges?

- How often does the team meet? Where? Was it more frequent meetings in the beginning?

- Do you interact with leaders at your VA on behalf of the lung cancer screening program? If so, how often? What type of communication (in-person meetings, emails, etc)? What is typically discussed?

- Do you interact with leaders at the VISN level? Does lung cancer screening seem like a priority of your VISN? If so, please describe.

8. **What has been difficult in implementing a lung cancer screening program?** *(ask this question if they have been involved in implementing a program from #5 above)*

**What do you think or anticipate would be some of the barriers to implementing a lung cancer screening program?** *(ask this question if they have NOT been involved in implementing a program from #5 above)*

- Do you have any experience in implementing a program in the VA? (if struggling to answer question)

- Obtaining buy-in from primary care, creating a multidisciplinary team multiple, gaining buy-in from leadership, coordinating Veteran care, providing smoking cessation, [who offers the smoking cessation?], tracking screening results and who is screened, etc.

9. **What has been difficult in screening Veterans from rural areas? Do you know of any patient barriers that would make it difficult for screening Veterans from rural areas, at least from your clinical experience?**

Possible Patient barriers: reaching patients, fear, anxiety, financial costs, lack of awareness of screening, doesn’t think it will help [e.g., trust that test is useful, trust in health system], transportation

Possible Healthcare system barriers: [I understand that a lot of steps are done for tools for communication, electronic tools like telehealth] insurance, availability of follow up appointment times for the follow-up procedures and does your local VA offer that or do they need to go to another VA that offers that service; electronic tools, workflow, communication, appointment time [what about rural provider awareness- word without sounding insulting]

10. *Program Directors Only*: **Is the navigator in your program a permanent position? Have you had any challenges with making this position permanent?** If yes, please describe (data will only be reported as aggregate and will be de-identified)

11. ***What could help you perform lung cancer screening?*** *(providers)*

**What kinds of changes in the current support system could help you?** *(staff/admin)*

12. **What do you think could help screen rural Veterans?**

[start broad and then go to rural] Provider level: educational materials – describe type website, posters, handouts, video for waiting room,

-[helpful to have educational materials to providers or patients- Provider: If lecture, describe preferences on amount of time and type of lecture – webinar, grand rounds, multidisciplinary, CME. application for handheld electronic, lecture.

-provider/patient reminder systems (describe preferences) how long should it be and how often should it be offered?

-System level: changes in information systems or electronic records systems, leadership, policies, other?

-[any difference for rural area] unique to the rural- talk about a lot of barriers in general to lcs-[make not redundant- eg. We’ve talked a lot about barriers specific to rural and are any other barriers that are unique/specific to the rural population?]

13. **How well does lung cancer screening fit with the existing work processes and practices in your VA?** *(providers)*

-What are likely issues or complications that may arise? [e.g. streamlining and integrated into workflow]

14. **Are meetings held regularly to discuss work processes and practices such as lung cancer screening?**

-Do you have a steering committee or regular meetings to discuss how things are going with lung cancer screening? [interested if other sites have a steering committee]

If they know they have a steering committee then ask:

Do you typically attend?

Do you know who typically attends these meetings?

What proportion of those expected to attend typically attend?

How often are the meetings held?

What is a typical agenda? How helpful are these meetings?

15. **What is currently working well with lung cancer screening at your VA? What is working well for screening Veterans who live in rural areas? What could work better?** *(staff/admin)*

[stay with broad until ready to specific to rural]

-Workflow, coordination, communication, increase in demand/radiology appointments, smoking history availability in chart, smoking cessation, shared decision-making documentation

16. **What are your hopes for the future of lung cancer screening?** **What could help maintain the program?** *(staff/admin)*

-provide excellent medical care to Veterans, cost effectiveness, team-based approach to providing high-quality care

As a non-MD (or someone who is not involved in direct patient care) is there anything I did not think to ask that you think would be helpful

Thank you very much for sharing your thoughts and experiences with us. We are learning a lot about this new screening test and your perspective is incredibly valuable as we aim to improve lung cancer screening implementation in the VA.

Supplementary Appendix S2

Coding System

| **Code** | **Label** | **Description** | **Notes** |
| --- | --- | --- | --- |
| **1** | **Screening Activity** | **Screening activity description** |  |
| 1.1 | Identify and initiate contact | Identification of patients who are appropriate for screening (older persons, heavy smokers) & following up with an outreach process |  |
| 1.2 | Shared decision making | Providers and patients discuss the benefits and risks of screening prior to making a decision (shared decision-making) |  |
| 1.3 | Test ordered | Provider orders screening test or refers the patient for screening |  |
| 1.4 | Smoking cessation offered | Smoking cessation services are offered |  |
| 1.5 | Coordinate_schedule appointment | Appointments for smoking cessation and screening are scheduled for the patient/s |  |
| 1.6 | Complete scan | Screening study (CT scan) is performed holistically, with adequate data collected |  |
| 1.7 | Radiology interpretation | Radiologist interprets the screening study & provides a comprehensive summary |  |
| 1.8 | Interpretation sent to referring provider | Screening results, along with data interpretation, are sent to the referring provider |  |
| 1.9 | Present results to patient | Referring provider discusses the screening results with the patient |  |
| 1.10 | Track and screen | Results are tracked over time in an electronic software system |  |
| 1.11 | Follow up | Annual or timely screening follow up or further evaluation is scheduled |  |
| 1.12 | Other screening activity | Other screening activity not listed above |  |
| **2** | **Lung cancer screening program characteristics** | **Discussion centers on characteristics of the lung cancer screening program** |  |
| 2.1 | Lung cancer screening program source | Discussion centers on how lung cancer screening program was developed (e.g., internally or externally) |  |
| 2.2 | Evidence Strength and Quality | Stakeholders’ perceptions of the quality and validity of evidence supporting the belief that the lung cancer screening program will have desired outcomes. |  |
| 2.3 | Complexity | Perceived difficulty of implementation, reflected by duration, scope, radicalness, disruptiveness, centrality, and intricacy and number of steps required to implement. |  |
| 2.4 | Relative advantage | Stakeholders’ perception of the advantage of implementing the lung cancer screening program versus an alternative solution. |  |
| 2.5 | Adaptability | The degree to which an lung cancer screening program can be adapted, tailored, refined, or reinvented to meet clinic or patient needs. | Also code if a suggestion relates to ways can be adapted |
| 2.6 | CT scan | Duration, structure, completeness |  |
| 2.6.1 | Features_structure | specific according to the intervention itself |  |
| 2.6.2 | Content_data | specific according to the intervention itself |  |
| 2.7 | Cost | Discussion centers on costs associated with implementation |  |
| 2.8 | Trialability | The ability to test the intervention on a small scale in the organization, and to be able to reverse course (undo implementation) if warranted. |  |
| 2.9 | Other program characteristic | Other lung cancer screening program characteristic not listed above |  |
| **3** | **Hospital_Organization setting** | **Discussion centers on clinic characteristics/Inner setting** |  |
| 3.1 | Structural characteristics | Clinic architecture, size, age, etc. |  |
| 3.2 | Clinic or organizational culture | Unique aspects of clinic (e.g., patient population, workflow, clinic norms/requirements/restrictions, protocols and processes) | What are likely issues or complications that may arise? [e.g. streamlining and integrated into workflow] |
| 3.3 | Implementation climate | Discussion centers on capacity for change, shared receptivity of the lung cancer screening program, etc. |  |
| 3.4 | Compatibility | The degree of tangible fit between meaning and values attached to the lung cancer screening program by involved individuals, how those align with individuals’ own norms, values, and perceived risks and needs, and how the lung cancer screening program fits with existing workflows and systems. |  |
| 3.5 | Relative priority | Individuals’ shared perception of the importance of the implementation within the organization. |  |
| 3.6 | Available resources | Possible resources dedicated for specifically for implementation (e.g., funding, staff retention) |  |
| 3.7 | Access to knowledge and information | Ease of access to digestible information and knowledge about the lung cancer screening program and how to incorporate it into work tasks. |  |
| 3.8 | Other inner setting | Other hospital or organizational discussion not listed above |  |
| **4** | **Outer Setting** | **CFIR outer setting constructs** |  |
| 4.1 | Patient Needs and Resources | The extent to which patient needs, as well as barriers and facilitators to meet those needs, are accurately known and prioritized by the organization. (e.g., social determinants/disparities) |  |
| 4.2 | Cosmopolitanism | The degree to which an organization is networked with other external organizations (local and national/global) |  |
| 4.3 | Clinical Informatics_Health Information Technology | Focus on the degree to which application of data analytics and technology are utilized to improve organization characteristics (as it pertains to health information data) |  |
| 4.4 | Pressure to implement | Mimetic or competitive pressure to implement an lung cancer screening program; typically because most or other key peer or competing organizations have already implemented or are in a bid for a competitive edge. |  |
| 4.5 | Guidelines_recommendations | Knowledge/utilization of guidelines, recommendations, or other decision-making tools |  |
| 4.6 | Other outer setting | Other outer setting discussion not listed above |  |
| **5** | **Individual characteristics** | **Constructs related to behaviors, attitudes, beliefs, and motivation of clinical team** |  |
| 5.1 | Knowledge_familiarity with the facts | Knowledge and familiarity with the lung cancer screening program |  |
| 5.1.1 | Knowledgeable | understanding of situation at hand |  |
| 5.1.2 | Limited_insufficient | (note: some interviewees did not know what the lung cancer screening program was about, although was under implementation) |  |
| 5.2 | Organizational specific beliefs | Perceived behavioral expectations associated with the lung cancer screening program. Also includes perception of other people's behaviors associated with the problem or the lung cancer screening program. |  |
| 5.2.1 | Descriptive | Perception of the behaviors of other people around you; what people typically do |  |
| 5.2.2 | Injunctive | Behaviors one is perceived to be expected to perform and also expects others to perform |  |
| 5.3 | Control beliefs | The extent to which one's beliefs control themselves/to which one exhibits beliefs that are characteristic of exhibiting control in different areas |  |
| 5.3.1 | Self efficacy | Ability to engage in the lung cancer screening program (e.g., belief in one's own ability to perform a behavior) |  |
| 5.3.2 | Individual stage of change | Stage of the individual (e.g., enthusiastic about lung cancer screening program) |  |
| 5.4 | Identification with organization | A broad construct related to how individuals perceive the organization, and their relationship and degree of commitment with that organization. |  |
| 5.5 | Program concerns | Specific concern related to the lung cancer screening program or implementation of the program |  |
| 5.6 | Anticipated outcomes | Discussion centers on participant's anticipated outcomes of the program (e.g., likely issues or complications that may arise) |  |
| 5.7 | Causal attributions | Discussion centers on what the participant associates as a cause for a given problem, success, etc. |  |
| 5.8 | Social Comparison | Discussion centers on individual drive to gain accurate self, patient, or institutional evaluations. Also comparison to other screening types |  |
| 5.9 | Experience with similar programs | Discussion centers around an experience with a similar program(s) |  |
| 5.10 | Preferences | Any discussion associated with a greater liking to one option over another |  |
| 5.11 | Other individual characteristic | Other individual characteristic not listed above |  |
| **6** | **Patient factors** | **Discussion centers on specific patient factors** |  |
| 6.1 | Knowledge and understanding | Pt knowledge and understanding about lung cancer screening |  |
| 6.2 | Symptoms | Any physical or mental feature which is regarded as indicating a condition of disease, particularly such a feature that is apparent to the patient. |  |
| 6.3 | Co_morbidity | Discussion centers on the simultaneous presence of two or more diseases or medical conditions in a patient. |  |
| 6.4 | Health behaviors | Patients health behaviors |  |
| 6.4.1 | Healthy | Engages in healthy behaviors (e.g., healthy diet) |  |
| 6.4.2 | Not healthy_harmful | Engages in unhealthy behaviors (e.g., smoking) |  |
| 6.5 | Environment | Discusses patient's home/city environment, etc. |  |
| 6.5.1 | Rural | in, relating to, or characteristic of the countryside rather than the town. | |
| 6.5.2 | Urban | in, relating to, or characteristic of a town or city. | |
| 6.6 | Insurance coverage | Discussion centers on insurance status or coverage |  |
| 6.7 | Logistical factors | E.g., access to reliable transportation |  |
| 6.8 | SES_financial situation | socioeconomic status and other financial factors that influence the patient's financial status (income, family support, insurance, etc.) |  |
| 6.9 | Competing priorities | Priorities such as family engagement, limited time, etc. |  |
| 6.10 | Intrapersonal factors | e.g., motivation, self efficacy, emotions and coping |  |
| 6.11 | Interpersonal factors | e.g., social support networks |  |
| 6.12 | Military service | Any discussion related to patient military service |  |
| 6.13 | Other patient factor | Other patient factor not listed above |  |
| **7** | **Communication** | **Discussion centering on the quality of communication** |  |
| 7.1 | Between providers | Discussion centers on communication between providers |  |
| 7.2 | Interprofessional engagement | Knowledge of interprofessional roles and responsibilities and utilizing the skills of the interprofessional team |  |
| 7.3 | Between patients and providers | Discussion focuses on the communication that occurs between patients and providers |  |
| 7.4 | Communication Modalities | Discussion centers on mode of communication; methods/information sources |  |
| 7.4.1 | In person | Discussion centers on in-person communication |  |
| 7.4.2 | EMR basket_note | Discussion centers on provider/interprofessional communication by way of EMR, notes, etc. |  |
| 7.4.3 | Telephone | Discussion between providers via phone |  |
| 7.4.4 | Telehealth | Discussion centers on patient communication through telehealth |  |
| 7.4.5 | Other communication modality | Discussion centers on other forms of communication |  |
| 7.5 | Goals and feedback | The degree to which program goals are clearly communicated, acted upon, and fed back to staff, and alignment of that feedback with goals. |  |
| 7.6 | Quality of communication | Any discussion related to the efficacy and clarity of communication |  |
| **8** | **Barriers_Facilitators** | **Participant describes a potential challenges or facilitators to successfully implement the program** |  |
| 8.1 | Barriers_challenges | Any event/situation that lead to a breakdown in the process of program implementation and/or screening protocol. |  |
| 8.1.1 | Systems level | Any discussion of a challenge/barrier involved systemic level issues including but not limited to buy in, funding, interfacility communication |  |
| 8.1.2 | Patient level | Any challenge/barrier involved patient issues including but not limited to anxiety, transport, education etc. | Will co-occur with patient factors category 6 |
| 8.2 | Facilitators | Any discussion of factors that enabled program implementation and/or screening protocol success |  |
| 8.3 | Contingency_conditional | Participant's response is contingent on a given factor |  |
| **9** | **Specific examples** | **Specific examples of situations/instances** | **Can also include anecdotal examples** |
| 9.1 | Went_going well | program implementation was successful/is going well |  |
| 9.2 | Error of omission | Program implementations that were excluded or unsuccessful |  |
| 9.3 | Error of commission | Program implementations unsuccessful due to implementation or screening errors |  |
| 9.4 | General example | Example not specified as went well/did not go well |  |
| 9.5 | Not observed | Participant states that s/he as not experienced a specific topic |  |
| **10** | **Process** | **Describe the process your VA went through to start a lung screening program. If you are currently in the decision-making process, what considerations have come up?** | **CFIR Constructs** |
| 10.1 | Planning | The degree to which a scheme or method of behavior and tasks for implementing an lung cancer screening program are developed in advance, and the quality of those schemes or methods. |  |
| 10.2 | Engaging | Attracting and involving appropriate individuals in the implementation and use of the lung cancer screening program through a combined strategy of social marketing, education, role modeling, training, and other similar activities. |  |
| 10.3 | Opinion leaders | Individuals in an organization who have formal or informal influence on the attitudes and beliefs of their colleagues with respect to implementing the lung cancer screening program. |  |
| 10.4 | Formally appointed internal implementation leaders | Individuals from within the organization who have been formally appointed with responsibility for implementing an lung cancer screening program as nurse navigator, coordinator, project manager, team leader, or other similar role. | Discusses the role and responsibility for screening activity and overall program responsibilities; Who would be responsible for ensuring that the lung cancer screening is implemented correctly and consistently over time? |
| 10.5 | External change agents | Individuals who are affiliated with an outside entity who formally influence or facilitate lung cancer screening program decisions in a desirable direction. |  |
| 10.6 | Executing | Carrying out or accomplishing the implementation according to plan. |  |
| 10.7 | Reflecting and evaluating | Quantitative and qualitative feedback about the progress and quality of implementation accompanied with regular personal and team debriefing about progress and experience. |  |
| **11** | **Suggestions and Needs** | **What kinds of changes in the current support system could help you? (will likely co-occur with other codes)** |  |
| 11.1 | Systems level | Systems-level suggestions and needs (e.g., changes in information systems or electronic records systems, leadership, policies) |  |
| 11.2 | Patient level | Discussion centers on patient-level changes |  |
| 11.3 | General suggestion | Suggestion that is not specific to the health system or the patient |  |
| **12** | **Provider_health team member** | **Discussion centers on the provider or health team member of discussion; role/specialty** |  |
| 12.1 | Radiology | Interactions with those in the radiology department |  |
| 12.2 | Pulmonology | Interactions with those in the pulmonology department |  |
| 12.3 | Primary care | Interactions with those involved in primary care |  |
| 12.4 | Oncology | Interactions with the oncology department |  |
| 12.5 | Surgery | Interactions with those involved in surgery |  |
| 12.6 | Navigator_coordinator | Interactions with navigators/coordinators |  |
| 12.7 | Nurse | Interactions with nurse | Do not code for nurse navigator |
| 12.8 | NP_PA | Interactions with nurse practitioner or physician assistant |  |
| 12.9 | Technician | Interactions with technicians |  |
| 12.10 | VISN | Interaction with VISN |  |
| 12.11 | Other provider or team member | interactions with other provider/health team members |  |
| **13** | **Practice_work experience** | **Designated columns** |  |
| 13.1 | Years of practice | How long have you been a __________(provider/staff/administrator) in _________ (specialty field)? | Enter as text- we will compute variable after coding |
| 13.2 | Other in last 5 years | Where else have you practiced/worked in the past 5 years? | Enter as text- we will compute variable after coding |
| 13.3 | Rural veteran percentage | percent of clinical practice live in rural area | Enter as text- we will compute variable after coding |
| 13.4 | Decision to implement personal involvement | Were you involved in or are you currently involved in the decision to implement a lung cancer screening program at your VA? |  |
| 13.4.1 | Involved in *decision* to implement | Were you involved in the decision to implement a lung cancer screening program at your VA? If yes, how were you involved? |  |
| 13.4.2 | Not involved in the *decision* to implement | Why were you not involved in the decision to implement a lung cancer screening program at your VA? |  |
| 13.5 | Status of implementation | Implemented/planning to implement |  |
| 13.5.1 | Implemented | lung cancer screening program is implemented |  |
| 13.5.2 | Planning to implement | Are you planning to implement a lung cancer screening program at your VA? |  |
| 13.6 | Other practice_work experience | Other practice or work experience not listed above |  |
| **14** | **World events** | **World events such as COVID-19 or political climate** |  |
| **15** | **Change over time** | **Discussion centers on change over time (e.g., Program evolve, VA quality of care)** |  |
| **16** | **Notable quotes** | **Quotes that stand out as having especially noteworthy content** |  |
| **5** | **Strategies and solutions for participation** | **Discussion centers around strategies used to educate the patient about the program** | **These codes apply when the discussion centers around these topics, can be both positive and negative** |
| 5.1 | Methods/information sources | Methods of education about the program |  |
| 5.1.1 | In person | Program education occur in person with provider/health team |  |
| 5.1.2 | Letter | Program education occur by way of postal mail |  |
| 5.1.3 | Monitor/television | Program education occur by way of waiting room television monitors |  |
| 5.1.4 | Posters | Program education occur by way of posters at the VA |  |
| 5.1.5 | Phone | Program education occur via phone call |  |
| 5.1.6 | My Healthy Vet | Program education occur via My Healthy Vet |  |
| 5.1.7 | Testimonials | Program education by way of testimonials |  |
| 5.1.8 | Other strategy or solution | Other strategy or solution |  |
| 5.3 | Shared decision making | Program enrollment involving shared decision making as strategy (intended to overcome barriers) |  |
| 5.4 | Setting expectations | Education includes setting realistic expectations |  |
| 5.5 | Resource coordination | Factors related to resource coordination (e.g., travel pay, screening site) |  |
| 5.6 | Rapport/trust/transparency/honesty | Individual mentions that creating a relationship between patient and provider helps create successful education and decision making |  |
| 5.7 | Appearance of presenter | Discusses demographic of person delivering information (if/how it matters) E.g., demographic, demeanor, etc. |  |
| 5.8 | Other strategy or solution/preference | Other strategy or solution/preference |  |
| 6.5 | Motivation to engage | Discussion centers on motivation to participate |  |
| 6.5.1 | Intrinsic | Discussion centers on intrinsic motivators (e.g., altruism) | |
| 6.5.2 | Extrinsic | Discussion centers on extrinsic motivators (e.g., better outcomes) | |
